# Mutant mouse models implicate a role for mGluR1/5, prolyl isomerase (Pin1) and Homer1a interactions in wakefulness

**DOI:** 10.3389/fnins.2025.1572258

**Published:** 2025-09-11

**Authors:** Brendan T. Keenan, Ewa Strus, Raozhou Lin, May Chan, Jie Lian, Raymond Galante, Paul Worley, Allan I. Pack, Nirinjini Naidoo

**Affiliations:** ^1^Division of Sleep Medicine, Department of Medicine, Perelman School of Medicine, University of Pennsylvania, Philadelphia, PA, United States; ^2^Department of Neuroscience, Johns Hopkins University, Baltimore, MD, United States; ^3^Chronobiology and Sleep Institute, Perelman School of Medicine, University of Pennsylvania, Pennsylvania, PA, United States

**Keywords:** sleep, Homer1a, wake, mGluR, Pin1

## Abstract

**Introduction:**

Healthy sleep and wake are integral to good health and occur when an organism is able to maintain long bouts of both sleep and wakefulness. Homer proteins have been shown to be important for sleep in both *Drosophila* and mice. For example, genetic deletion of *Homer1a* in mice results in failure to sustain long bouts of wakefulness. Homer1a has also been shown to amplify mGluR activity by facilitating binding of the prolyl isomerase Pin1 to mGluR. This study uses mouse models to evaluate whether the *Homer1a* null sleep phenotype may be dependent on the mGluR-Pin1 interaction and examines sleep/wake behavior.

**Methods:**

EEG recordings were used to determine and compare sleep and wake in three different mouse models and their littermate control mice. Mouse models included: mGluR(TS-AA) knock-in mice in which Pin1 binding is prevented and activity-dependent prolyl isomerization of mGluR is inhibited; mGluR(F-R) knock-in mice in which Homer binding is eliminated but Pin1 binding is allowed; and a Homer1a null, mGluR(F-R) double mutant mouse to evaluate whether Pin1 binding can rescue the Homer1a knock-out phenotype. Sleep-wake behavior was analyzed using traditional summary measures and a spike-and-slab mixture distribution to better characterize microarchitecture.

**Results:**

Knock-in mGluR(TS-AA) mice display a reduced ability to sustain long bouts of wakefulness during the active lights off period, recapitulating part of the previously observed wake phenotype of the *Homer1a* knock-out mouse. Alteration of the Homer binding site to mGluR in mGluR(F-R) knock-in mice has no effect on the sleep phenotype, whereas crossing the mGluR(F-R) knock-in into the *Homer* null background resulted in increased duration of long wake bouts, suggesting a restored ability to maintain wakefulness, with other sleep/wake characteristics similar to littermate mice.

**Conclusion:**

These studies highlight the role of Pin1 binding to mGluR as a potential mechanism in the control of sleep/wake behavior. Future studies should explore whether other binding partners of Homer and mGluR also affect sleep and wake.

## Introduction

One of the postulated functions of sleep is to modulate synaptic scaling and plasticity (Benington and Frank, 2003; Wang et al., 2011). Although data exists on how synaptic transcripts and protein expression and localization change during sleep, less is known about how these plasticity-related proteins interact to regulate sleep and wake. One such family of proteins, Homer, is thought to be directly involved in sleep homeostasis and sleep/wake regulation. Homer proteins function as molecular adaptors binding Group I metabotropic glutamate receptors (mGluR) via a proline-rich motif to ionotropic and Ca^2+^ signaling receptors at the post-synaptic density (Xiao et al., 1998). Constitutively expressed Homer proteins (Homer1b/c, Homer2, and Homer3) self-multimerize via their C-terminal coiled-coil regions to create these crosslinking scaffolds (Xiao et al., 1998) and bind to other interacting proteins via the N-terminus EVH1 domain. These scaffolds are disrupted by the dominant negative form of Homer, Homer1a, which lacks the C-terminal coiled-coil domain. Because of this alternative splicing, Homer1a is a short form protein while Homer1b/c, Homer2, and Homer3 with the C-terminal coiled-coil domain are classified as long form Homer proteins. Homer1a is upregulated in response to neural activity (Brakeman et al., 1997).

Previous studies have demonstrated that *Homer1a* is one of a number of genes differentially expressed in response to sleep deprivation in several mouse strains (Mackiewicz et al., 2007; Maret et al., 2007). Bioinformatics analyses of the increase in delta power in slow wave sleep following sleep deprivation identified *Homer1a* as the strongest sleep homeostasis candidate (Mackiewicz et al., 2008). However, in subsequent studies we found no difference in the delta power response to sleep deprivation in *Homer1a* knock-out (KO) mice compared to wildtype mice (Naidoo et al., 2012). Instead, *Homer1a* knock-out mice displayed an inability to maintain wakefulness, with shorter wake bouts during the active lights off period (Naidoo et al., 2012), suggesting a role for Homer1a in the regulation of wakefulness. We also determined that in addition to increasing in the cerebral cortex, Homer1a also increases in the claustrum with wakefulness (Zhu et al., 2020). Since Homer1a is not an output of the primary wake-promoting brain regions that typically inhibit sleep-promoting areas, its influence on sleep and wake likely occurs through a different mechanism than posited by the well-known flip-flop model (Saper and Fuller, 2017) which suggests that sleep and wake states are controlled by opposing, mutually inhibitory interactions between wake-promoting neurons (such as those that release norepinephrine, dopamine, orexin, and histamine) and sleep-promoting neurons located in the ventrolateral and median preoptic areas of the hypothalamus. The specific mechanism through which Homer1a contributes to sleep and wake remains to be determined. Mechanisms underlying the regulation and maintenance of wakefulness are key for the understanding of sleep disorders such as excessive daytime sleepiness, narcolepsy and hypersomnolence. An inability to maintain wakefulness is a core symptom of all these disorders (Mahoney et al., 2019; Trotti and Arnulf, 2021). The identification of a Homer1a-mGluR-Pin1 (protein interacting with NIMA 1) mechanism (Park et al., 2013) led us to the hypothesis evaluated in the present study – that the Homer1a sleep/wake phenotype may depend on the mGluR-Pin1 mechanism.

**Figure 1:**
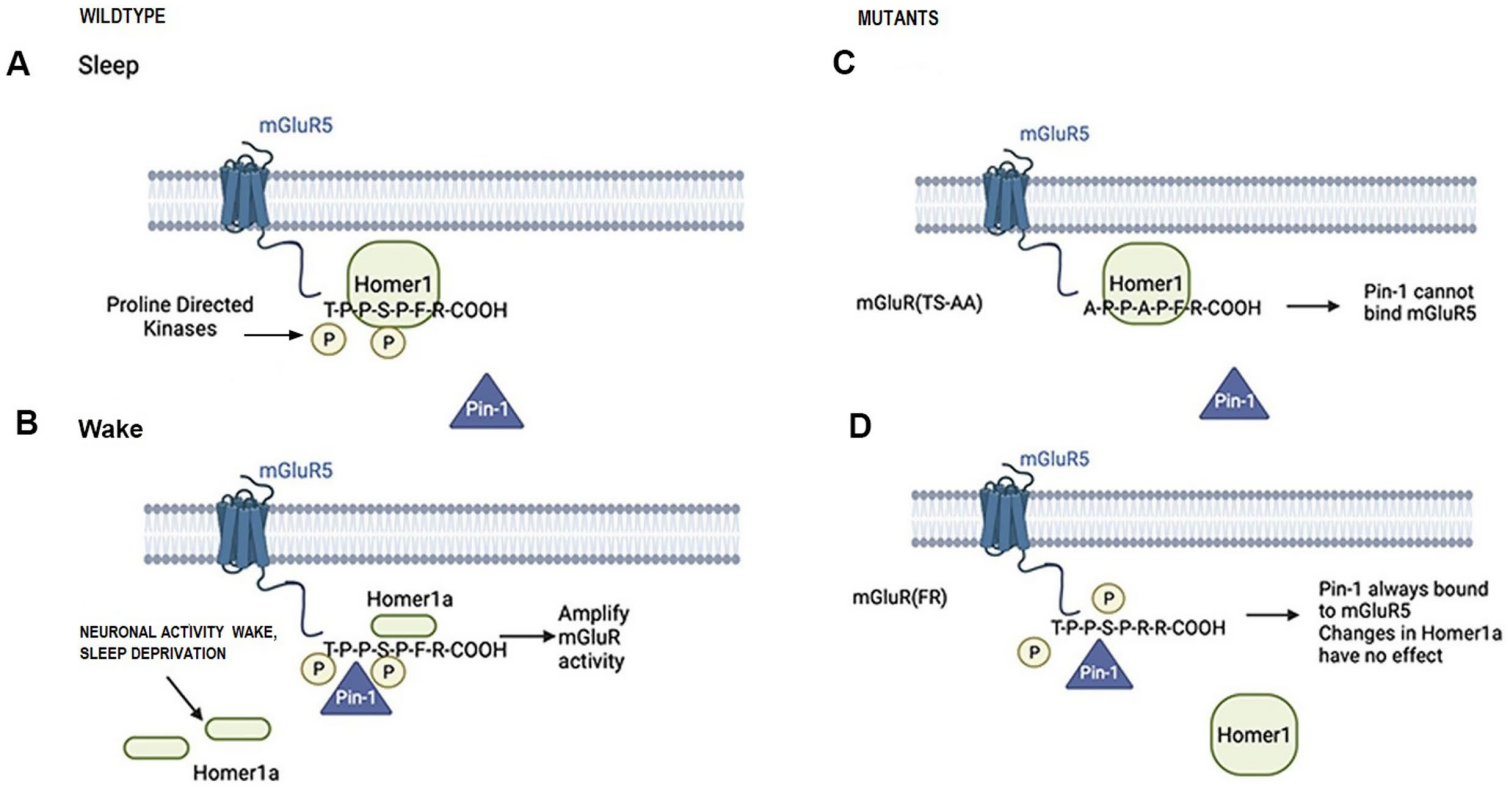
Schematic illustrations of Homer-mGluR and Pin1 proposed interactions during sleep (panel **A**) and waking (panel **B**) and in the mGluR(TS-AA) (panel **C**) and mGluR(F-R) (panel **D**) knock-in mice.

The EVH1 domain of Homer1 binds a consensus PPXXF sequence that is present in mGluR1/5, Shank, and Preso1 (Beneken et al., 2000). Homer1 crosslinking is in dynamic competition with Homer1a, which contains only the EVH1 domain and, thus, can bind to the same target proteins but does not self-associate. Homer crosslinking influences the signaling and pharmacology of mGluR1/5 (Ango et al., 2001; Hu et al., 2012). mGluR5 is a group I member of the metabotropic g-protein coupled receptor family that activates intracellular signaling cascades to modulate synaptic plasticity and neurotransmission (Conn and Pin, 1997). Furthermore, mGluRs have been implicated in sleep–wake regulation in rodents (Ahnaou et al., 2015; Pritchett et al., 2015). In addition, genetic knockdown of the single mGluR in *Drosophila* (*DmGluRA*) reduced daytime wakefulness (Ly and Naidoo, 2019). Homer1 and Homer1a also modulate mGluR5 phosphorylation and interaction with Pin1, a prolyl isomerase (see Figure 1) (Park et al., 2013). Full-length Homer1c and Pin1 both compete to bind to mGluR. Under conditions of increased neuronal activity and upregulated Homer1a expression, Homer1a displaces the full length Homer1c from mGluR, thereby facilitating Pin1 association and promoting enhanced mGluR signaling. Pin1 binds mGluR5 better in the presence of the short Homer1a (and not Homer1c; see Figure 1) (Park et al., 2013). Thus, we hypothesized that this amplification of mGluR activity would promote sustained or longer bouts of wakefulness.

To investigate this role for the mGluR-Homer-Pin1 dependent signaling pathway in sleep/wake regulation and explore whether this mechanism underlies the Homer1a knock-out phenotype, we used two mGluR5 knock-in mouse models that have wildtype mGluR5 replaced with mutant forms of mGluR5. The first knock-in mutant mouse, mGluR(TS-AA), has both the threonine and serine residues adjacent to the proline in the T-P-P-S-P-F binding motif replaced by alanine, thus eliminating potential phosphorylation sites. These mutations have been shown to prevent Pin1 binding (Park et al., 2013) and are expected to inhibit activity-dependent prolyl isomerization of mGluR (see Figure 1). Since this disables the mechanism involving Homer1a, we hypothesized that this mutant should have a similar sleep phenotype to the *Homer1a* knock-out. A second mutant mouse line, mGluR(F-R), has the Homer1 binding site P-P-X-X-F altered such that the phenylalanine at position 1,128 in the Homer binding domain is substituted with arginine, which eliminates Homer binding but allows Pin1 binding (see Figure 1). Since this allows normal Pin1 binding, we hypothesized that the sleep/wake phenotype in this mutant will be similar to that in wildtype mice. To further test whether the Pin1 mechanism was in part responsible for the *Homer1a* knock-out phenotype, we crossed the *Homer1a* knock-out mouse with the mGluR(F-R) mouse to generate a double mutant [Homer1a−/−; mGluR(F-R)] that we expected to rescue the sleep/wake phenotype found in the *Homer1a* knock-out. Finally, we carried out biochemical and molecular analyses to substantiate behavioral observations.

## Materials and methods

### Mice and breeding strategies

All experiments were performed on male mice at 11–13 weeks of age maintained on a 12-h light/dark cycle (lights on 0700; 80 Lux at the floor of the cage) in a sound attenuated recording room with a temperature of 22–24 °C. Food and water were available *ad libitum*. Animals were acclimated to these conditions for 10–14 days before beginning any studies. All animal experiments were performed in accordance with the guidelines published in the NIH Guide for the Care and Use of Laboratory Animals and were approved by the University of Pennsylvania Animal Care and Use Committee. The mGluR(TS-AA) and mGluR(F-R) mice were created as described previously (Park et al., 2013). Breeding pairs of each of the knock-in mice were obtained from the Worley laboratory. Homer1a−/−;mGluR(F-R) double mutants were generated by crossing mGluR(F-R) with Homer1a KO mice. F1 heterozygous mice were crossed to generate F2 Homer1a KO: mGluR(F-R) double mutants and wildtype littermates used in behavioral studies. Mice genotypes were confirmed using the following primers: mglur 117: 5′- AAG CAT TCA AGG CCA TAC AC -3′; mglur 481: 5′- AGG GAG GAA GAG GTG GAA GA − 3′; and mglur 615: 5′- TGC AAA TGT GGA GGT TGG TA − 3′. The Homer1a primers for genotyping the double mutant were as described in Naidoo et al. (2012) and are listed below as well.

H1aB2: 5’-AGTCAAAGAGGTCCCTCTGTTCTTG-3′ (reverse).

H1aB3: 5’-TCATGTTTACAGTCCAGTAATGCC-3′ (reverse).

H1aA3:5’-TGTGACACAGAACTCAGCCAAG-3′ (forward).

### EEG/EMG recording of sleep and scoring of sleep/wake and sub-stages of sleep

Sleep/wake behavior was recorded via EEG/EMG as previously described (Pack et al., 2007; Naidoo et al., 2018). Wake, non-rapid eye movement (NREM) and rapid eye movement (REM) sleep were manually scored as described previously (Pack et al., 2007) in 4-s epochs during 24-h baseline recordings. Baseline spectral changes were determined as described by Hasan et al. (2012). Briefly, EEG spectra were expressed individually as a percentage of the average power of frequencies between 0.25 and 30 Hz. Sleep and wake spectra were assessed from the 12-h baseline lights-on or lights-off recordings, respectively.

### Sleep deprivation

For biochemical/molecular analyses, mice were sleep deprived for 1, 3 or 4 h starting at 7 AM. Deprivation was performed through gentle handling (Pack et al., 2007), following an acclimation period for handling procedures.

### Western analyses

Brain cortical tissue from undisturbed or sleep deprived mice were homogenized and analyzed by SDS gel electrophoresis and western blotting as previously described (Naidoo et al., 2018). Antibodies used were as described below.

### Antibodies

The mGluR5 phospho-S1126 and mGluR5 phosphoT1123 antibodies are from the Worley lab and are described previously (Park et al., 2013). All other antibodies were acquired commercially and their dilutions are: mGluR5 (Upstate) at 1:10000, Pin1 (Upstate) at 1:3000, GluA1 (JH1710) at 1:1000, GluA2 (JH1707) at 1:500, NR1 (Millipore) at 1:1000, pan Homer at 1:10000, Actin (Sigma Aldrich) at 1:10000, and Homer1a (Synaptic Systems-currently discontinued).

### Sleep and wake phenotypes

Traditional summaries of sleep and wake, including total duration (minutes), number of bouts, and average bout duration (minutes) of wake, NREM and REM sleep (as well as total sleep), were summarized over 24 h and separately during lights on (7 AM-7 PM) and lights off (7 PM-7 AM). To provide more detailed insights into the microarchitecture of sleep and wake, we also applied our previously-described spike-and-slab phenotyping methodology shown to reveal greater differences between mouse strains (McShane et al., 2010; Naidoo et al., 2018). Rather than traditional summaries of all wake, NREM or REM bouts for a given mouse, the spike-and-slab method utilizes a mixture distribution approach that separately describes the characteristics of short bouts (≤40 s; the “spike”) and long bouts (>40 s; the “slab”) for wake, NREM and REM bouts conditional on the preceding state. The “spike” is modeled as a set of 10 values (π_i_) equal to the probability that a given bout lasts exactly *i* epochs, while the “slab” is modeled using the *α* and *β* parameters of a gamma distribution. These parameters are used to generate the size of the “spike” (e.g., the proportion of short bouts) and the size of the “slab” (e.g., the average duration of long bouts); the total number of bouts is also generated. Of particular focus in the present manuscript are the characteristics of NREM bouts that were preceded by wake and wake bouts that were preceded by NREM; data on wake bouts preceded by REM are also summarized (McShane et al., 2010, Naidoo et al., 2018). Additional details on the underlying mathematical modeling and assumptions of the spike-and-slab approach are described by McShane et al. (2010) and Naidoo et al. (2018). Sleep and wake phenotypes were available on: (a) *n* = 7 mGluR(TS-AA) and *n* = 7 wildtype littermate controls; (b) *n* = 8 mGluR(F-R) and *n* = 8 wildtype littermate controls; and (c) *n* = 6 Homer1a−/−; mGlur(F-R) and *n* = 8 wildtype littermate controls.

### Statistical analysis

Data are summarized using means and standard errors. Comparisons between genotypes were performed using non-parametric Wilcoxon rank-sum tests given our small sample size and to limit possible impact of any deviations in the normality assumption. To understand the relative magnitude of observed differences, standardized mean differences (SMDs; or, equivalently, Cohen’s d) were estimated as the observed mean difference divided by the pooled standard deviation between genotypes; values of 0.2, 0.5 and 0.8 can be interpreted as small, moderate and large effects (Cohen, 1988). A *p* < 0.05 was reported as nominally significant, and a Hochberg correction (Hochberg, 1988; Huang and Hsu, 2007) was applied to determine statistical significance in the context of multiple comparisons within a given genotype and time-period of interest. Similar to our previous publication on *Homer1a* knock-out mice (Naidoo et al., 2012), to evaluate the differences in bout length distributions between wildtype and mutant mice, we generated empirical Q-Q plots and compared the observed distributions of sorted values in mutant vs. wildtype mice against 1,000 randomly generated null distributions from the observed data in wildtype mice; a significant difference in bout distributions would result in a line outside of the randomly generated null region.

A *post hoc* power calculation was performed to determine our ability to detect various effect sizes when comparing sleep/wake for each genotype versus wildtype littermate controls. At the observed sample sizes, our study had >80% power for large SMD of between 1.5–1.7, depending on the genotype. Specifically, for mGluR(TS-AA) (*n* = 7) vs. wildtype (*n* = 7), our study had 80% power to detect an SMD of 1.63 at an *α* = 0.05. For mGluR(F-R) (*n* = 8) vs. wildtype (*n* = 8), our study had 80% power to detect an SMD of 1.51 at an α = 0.05. For Homer1a−/−; mGlur(F-R) (*n* = 6) vs. wildtype (*n* = 8), our study had 80% power to detect an SMD of 1.65 at an *α* = 0.05. In addition to reporting statistical significance results are interpreted with respect to the strength of the observed effect sizes (Lederer et al., 2019; Wasserstein et al., 2019).

## Results

### mGluR(TS-AA) knock-in mice show a similar sleep/wake phenotype as seen in Homer1a knock-out mice

**Figure 2:**
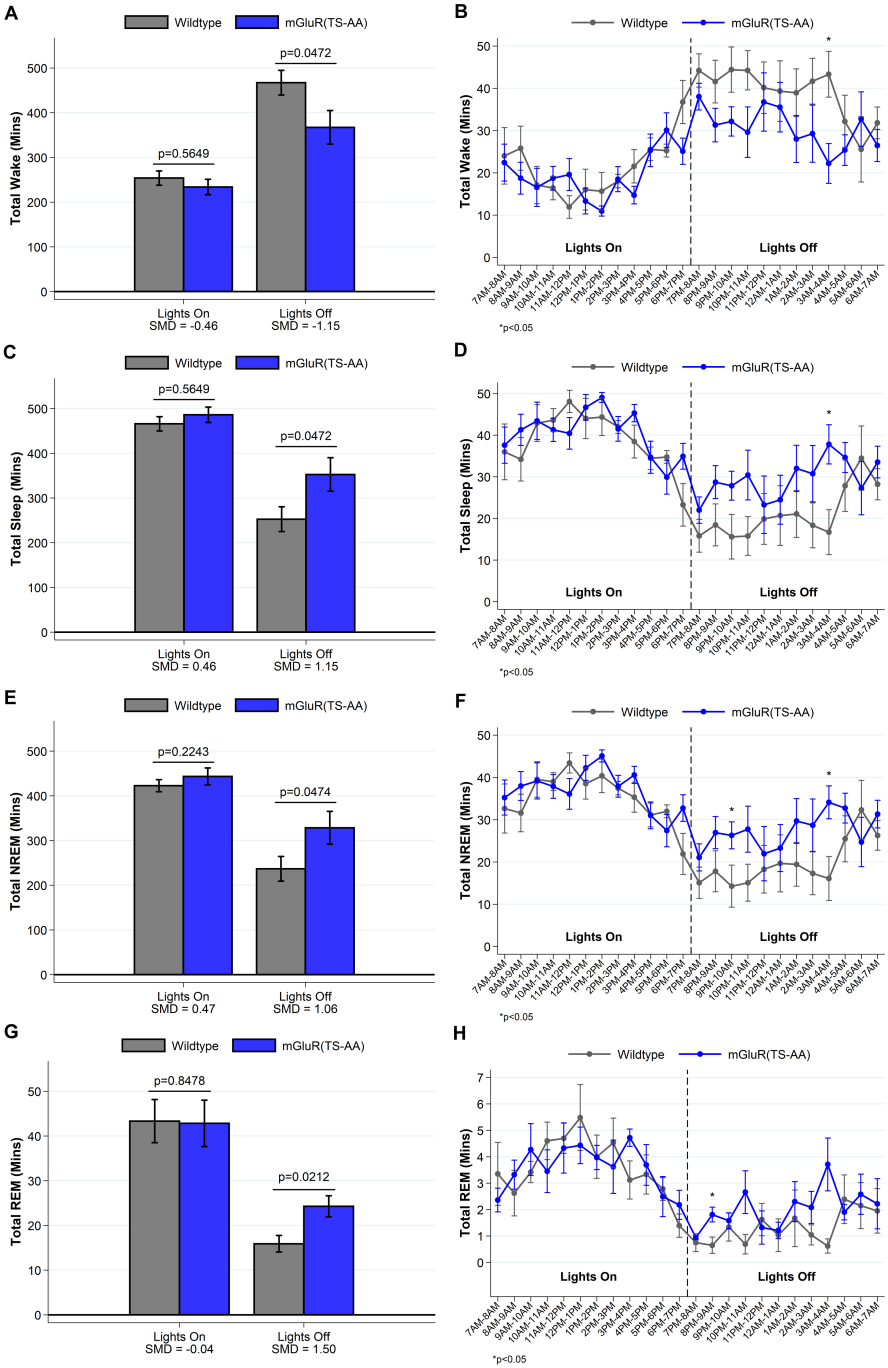
Total amounts of sleep and wake among mGluR(TS-AA) (*n* = 7) and wildtype littermate control (*n* = 7) mice. For lights on/off and per hour of recording, data on minutes of wake (panels **A**,**B**), and total (panels **C**,**D**), NREM (panels **E**,**F**) and REM (panels **G**,**H**) sleep are summarized. Consistent with our expectation that mGluR(TS-AA) mice will have a similar sleep/wake as observed in *Homer1a* KO mice, we see decreased wakefulness and increased sleep amounts in the mGluR(TS-AA) mice during the lights off period. Data are consistent when summarizing the entire lights off period or when comparing the hourly measures. Data presented as mean±SE.

**Figure 3:**
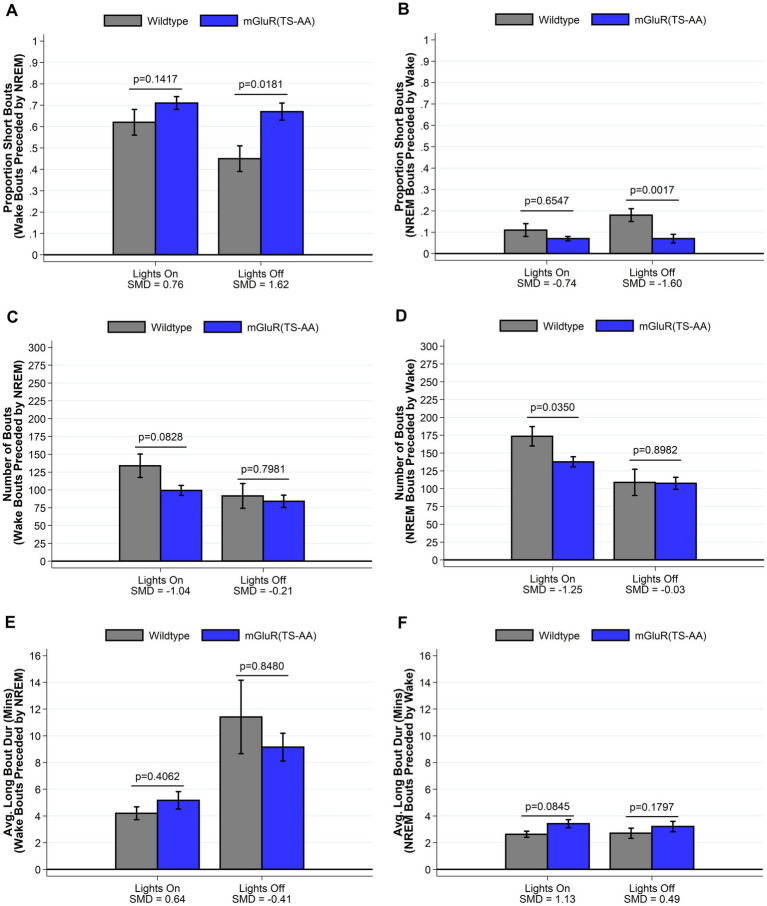
Sleep and wake phenotypes estimated from spike and slab approach in mGluR(TS-AA) (*n* = 7) and wildtype littermate control (*n* = 7) mice. Phenotypes describing the number (panel **A**), proportion of short (panel **C**), and duration of long (panel **E**) wake bouts preceded by NREM (e.g., primary wake bouts) and number (panel **B**), proportion of short (panel **D**), and duration of long (panel **F**) NREM bouts preceded by wake (e.g., primary sleep bouts) are illustrated in mGluR(TS-AA) and wildtype control mice. Consistent with our hypothesized reduced ability to maintain wakefulness, mGluR(TS-AA) mice show a greater estimated proportion of short wake bouts preceded by NREM and a smaller proportion of short NREM bouts preceded by wake during lights off. Data on the number of NREM bouts preceded by wake and the duration of long NREM bouts preceded by wake are consistent with more consolidated NREM sleep during lights on for mGluR(TS-AA) mice. Data presented as mean±SE.

We have previously shown that *Homer1a* knock-out mice display a reduced ability to maintain wakefulness, with shorter wake bouts during the active lights off period (Naidoo et al., 2012). Consistent with our hypothesis that mGluR(TS-AA) mutant mice would display a similar phenotype to *Homer1a* knock-out mice, during the lights off period we observed decreased wake and increased sleep amounts (see Figure 2) in the mGluR(TS-AA) mice compared to wildtype controls, as well as a greater proportion of short bouts of wake preceded by NREM and smaller proportion of short bouts of NREM preceded by wake (see Figure 3); there was a trending, but not statistically significant difference in average wake bout duration. Thus, supporting a role for the Pin1-mGluR5 binding mechanism in sleep/wake regulation, the mGluR(TS-AA) show a similar reduced ability to maintain wakefulness as seen in the Homer1a KO mice (Naidoo et al., 2012). Details are provided in the following sections, focused on key differences during the lights off (active) and lights on (sleep) periods; all data (including summarized over 24 h) is presented in Supplementary Tables S1 and S2.

#### mGluR(TS-AA) mice display a reduced ability to maintain wakefulness during the active lights off period

An examination of sleep and wake during the lights on and light off periods indicated that the mGluR(TS-AA) mice displayed significantly less wake and more sleep during the lights off period (*p* < 0.05; Figure 2 and Supplementary Table S1). As hypothesized, the mGluR(TS-AA) mice had ~100 min less wake on average (367.3 ± 37.6 min) compared to wildtype littermates (467.3 ± 27.6 min) during the lights off period (SMD = −1.15, *p* = 0.047; Figure 2A). We also observed a shorter average wake bout duration in lights off in mGluR(TS-AA) mice (3.72 ± 0.71 min) than in wildtype mice (6.37 ± 1.61), with a large effect size (SMD = −0.80), although the difference was not statistically significant (*p* = 0.064). The number of wake bouts (*p* = 0.848) did not differ from that in the wildtype littermates during the lights off period and the number and duration of sleep bouts were generally comparable.

**Figure 4:**
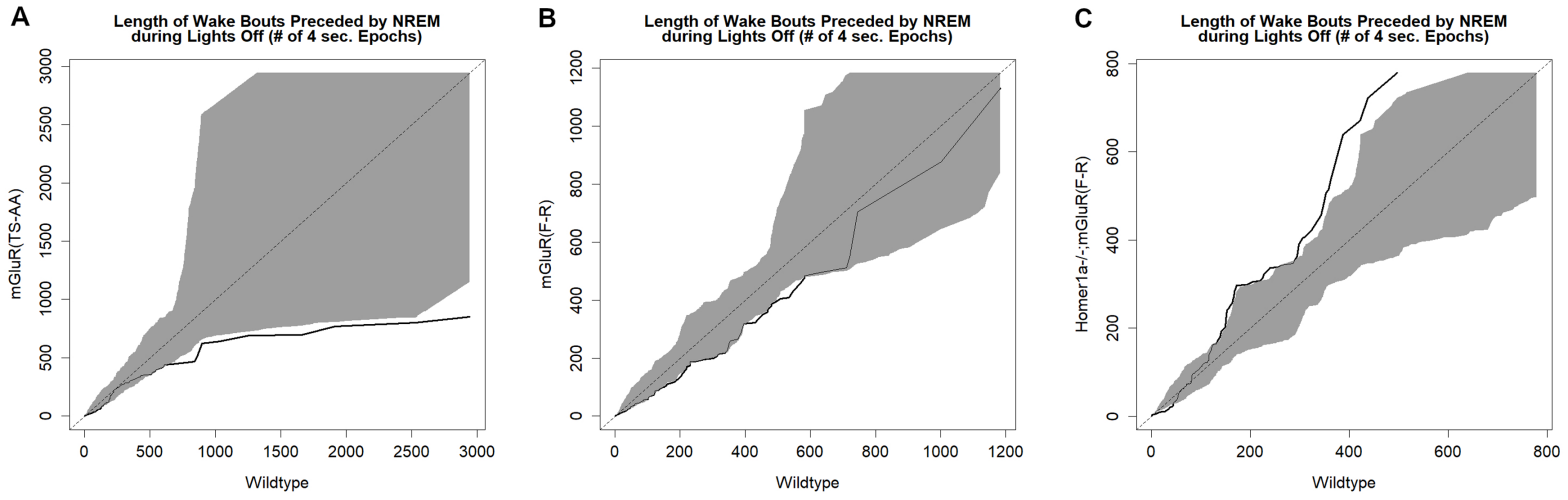
Empirical Q-Q plots illustrating the distribution of wake bout durations in each genotype. These plots illustrate the distribution of wake bout durations in 4 s epochs for mGluR(TS-AA) (panel **A**), mGluR(F-R) (panel **B**) and Homer1a −/−; mGluR(F-R) (panel **C**) mice on the Y-axis and wildtype littermates on the X-axis. Identical distributions would fall on the line of identify; the grey area on the plot is the estimated variance around the line of identity (null region). The Q-Q line is indicated in black. Results demonstrate reduced ability to maintain wakefulness in the mGluR(TS-AA) mice, as evidenced by the Q-Q line falling below the null region and a rescued wakefulness phenotype in the Homer1a −/−; mGluR(F-R) mice, as evidenced by the Q-Q line falling above the null region.

We next compared sleep/wake microarchitecture using the more detailed spike-and-slab phenotypes (see Figure 3 and Supplementary Table S2) (Naidoo et al., 2018; McShane et al., 2010). Consistent with the hypothesized reduced ability to maintain wakefulness within the mGluR(TS-AA) mice, there was a greater proportion of short bouts of wake preceded by NREM in the mGluR(TS-AA) compared to wildtype mice during lights off (0.67 ± 0.04 vs. 0.45 ± 0.06; SMD = 1.62; *p* = 0.018; Figure 3A and Supplementary Table S2). This result is also reflected in a *smaller* proportion of short bouts of NREM preceded by wake within the mGluR(TS-AA) mice during lights off (0.07 ± 0.02 vs. 0.18 ± 0.03; SMD = −1.60, *p* = 0.002; Figure 3B and Supplementary Table S2). To further illustrate this difference in the ability to maintain wakefulness during the lights off period, we generated empirical Q-Q plots comparing the distribution of bout lengths (quantified as number of 4-s epochs) in mGluR(TS-AA) and wildtype mice (see Figure 4A). The observed relative distribution of bouts of wake preceded by NREM durations falls outside of the null region, supporting significantly shorter bouts of this type among the mGluR(TS-AA) mice; this empirical Q-Q plot is very similar to the previous result in *Homer1a* knock-out mice (Naidoo et al., 2012). Together, these results support a consistent sleep/wake phenotype in mGluR(TS-AA) mice as previously observed in the *Homer1a* knock-out phenotype (Naidoo et al., 2012).

#### mGluR(TS-AA) mice display more consolidated NREM during the lights on (sleep) period

In addition to evaluating the hypothesized reduced ability to maintain wakefulness during lights off, results demonstrate more consolidated sleep in the mGluR(TS-AA) mice than in wildtype mice during the lights on period. Specifically, the mGluR(TS-AA) mice had fewer sleep (*p* = 0.035), NREM (*p* = 0.047) and wake (*p* = 0.035) bouts during the lights on period, as well as longer sleep (*p* = 0.064) and NREM (p = 0.035) bout durations using traditional summary measures (Supplementary Table S1). Similar results were observed with the spike-and-slab phenotypes, with mGluR(TS-AA) mice having fewer bouts of NREM preceded by wake (SMD *=* −1.25, *p* = 0.035; Figure 3D) and some evidence of longer duration of long bouts of NREM preceded by wake (SMD *=* 1.13; *p* = 0.085; Figure 3F) during lights on compared to wildtype mice (see also Supplementary Table S2).

#### mGluR(TS-AA) mice display similar EEG power spectra to wildtype mice

We next compared the EEG power spectra recorded during wake, NREM, and REM states between the mGluR(TS-AA) and the wildtype littermates (see Supplementary Figure S1). No significant differences were observed in the spectra during wake or NREM and REM sleep phases between the two genotypes. Total delta and theta power densities of the EEG were also calculated. The delta power density, which is considered to be an indicator of the homeostatic sleep need during NREM sleep (Franken et al., 2001) was not significantly different between the mGluR(TS-AA) and the wildtype littermates.

### mGluR(F-R) mice display a sleep phenotype more similar to wildtype mice

**Figure 5:**
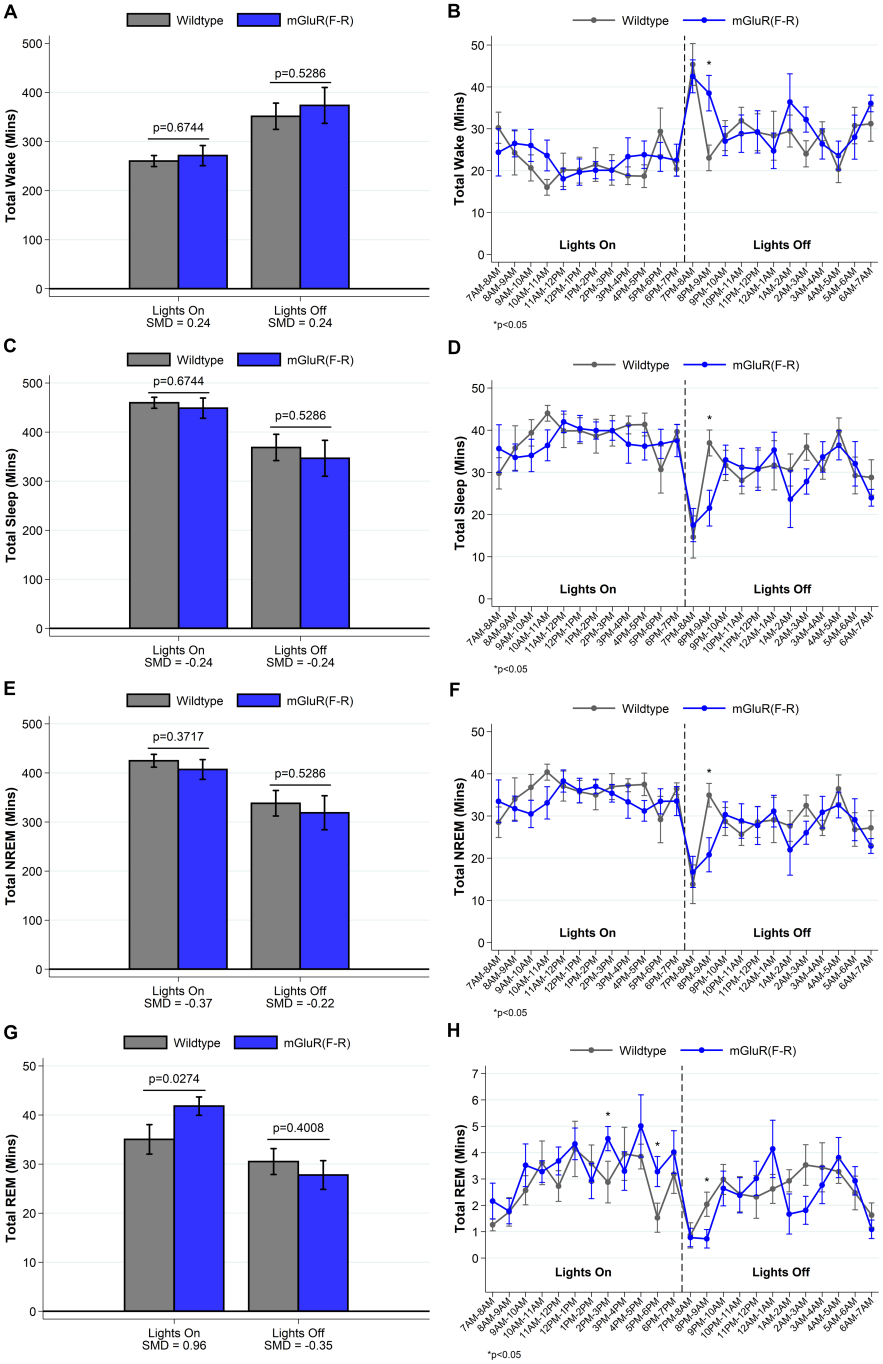
Total amounts of sleep and wake among mGluR(F-R) (*n* = 8) and wildtype littermate control (*n* = 8) mice. For lights on/off and per hour of recording, data on minutes of wake (panels **A**, **B**), and total (panels **C**, **D**), NREM (panels **E**, **F**) and REM (panels **G**, **H**) sleep are summarized. Consistent with expectations, we find no meaningful differences between mGluR(F-R) mice and wildtype mice in the amounts of sleep/wake during lights off. Data show a 7 min increase in the REM sleep during lights on within the mGluR(F-R) mutant mice (SMD = 0.96; *p* = 0.027), but no differences in the amounts of wake, total sleep or NREM sleep. Data presented as mean±SE.

**Figure 6:**
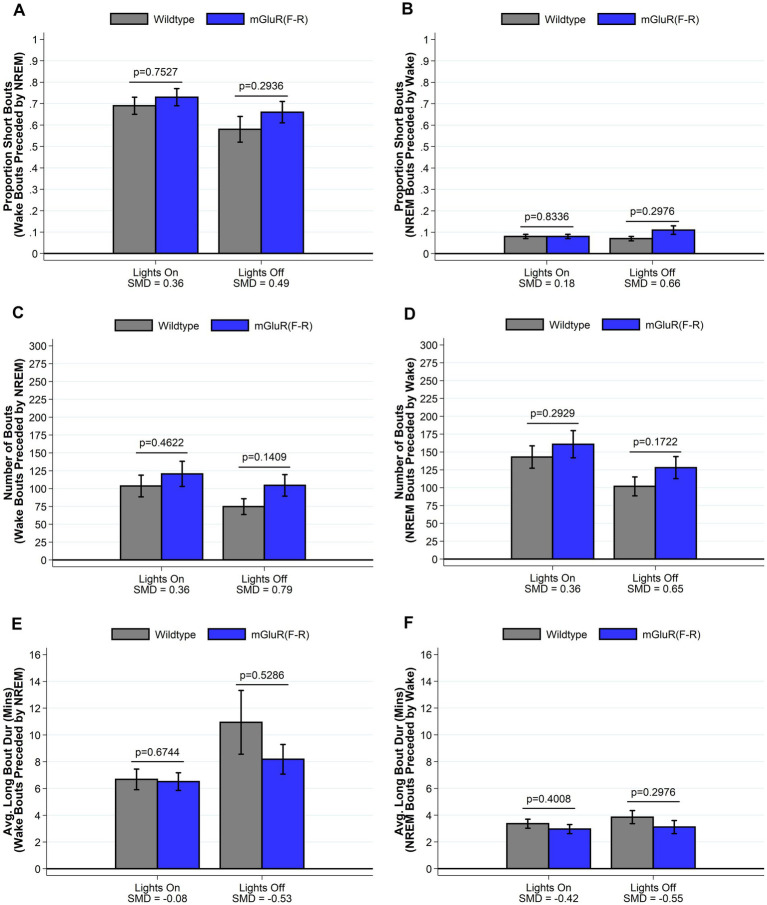
Sleep and wake phenotypes estimated from spike and slab approach in mGluR(F-R) (*n* = 8) and wildtype littermate control (*n* = 8) mice. Phenotypes describing the number (panel **A**), proportion of short (panel **C**), and duration of long (panel **E**) wake bouts preceded by NREM (e.g., primary wake bouts) and number (panel **B**), proportion of short (panel **D**), and duration of long (panel **F**) NREM bouts preceded by wake (e.g., primary sleep bouts) are illustrated in *mGluR(F-R)* and wildtype control mice. We find no meaningful differences between mGluR(F-R) mice and wildtype mice in sleep/wake microarchitecture. Data presented as mean±SE.

To test the hypothesis that the observed phenotype in the *Homer1a* knock-out and mGluR(TS-AA) mouse was dependent on *Pin1* binding, we performed similar sleep and wake phenotyping within the mGluR(F-R) mutant that eliminates Homer1 binding but allows Pin1 binding in mGluR (see Supplementary Tables S3 and S4). Consistent with our hypothesis, we found that the amounts of sleep and wake were similar between mGluR(F-R) mice and their wildtype littermates (see Supplementary Table S3 and Figure 5), apart from some evidence of more REM sleep during lights on in the mGlur(F-R) genotype (SMD = 0.96, *p* = 0.027; Figure 5G). Further, we found no significant differences in the numbers and duration of sleep and wake bouts between wildtype and mGluR(F-R) mice (Supplementary Table S3). Consistent with these results, no statistically significant differences were observed in sleep characteristics measured using the spike-and-slab approach (see Figure 6 and Supplementary Table S4). Notably, there was no difference in the proportion of short bouts of wake preceded by NREM in the mGluR(F-R) (Figure 6A), as was observed in the mGluR(TS-AA) and Homer1a knock-out mice, with the empirical Q-Q plot of the length of wake bouts preceded by NREM falling within the null distribution (see Figure 4B). Therefore, overall, we find that the mGluR(F-R) mutant mice are more similar to wildtype littermate controls than either the mGluR(TS-AA) or the previously described *Homer1a* knock-out, supporting a role of Pin1-mGluR interaction in the observed sleep/wake phenotypes.

### The reduced wake phenotype in Homer1a knock-out mice is not observed in the Homer1a-mGluR(F-R) double mutant that allows Pin1 binding to mGluR

**Figure 7:**
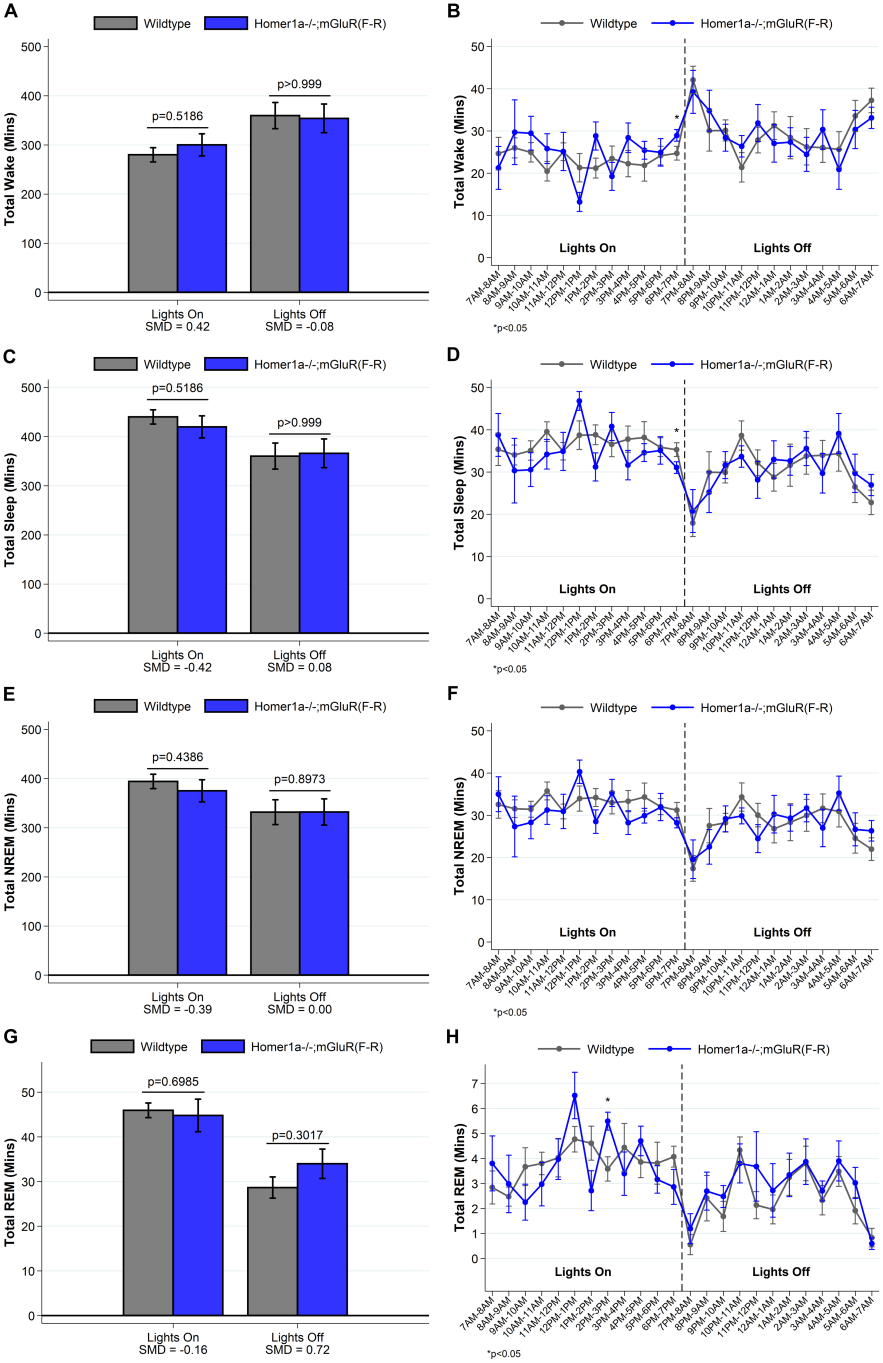
Total amounts of sleep and wake among Homer1a −/−; mGluR(F-R) (*n* = 6) and wildtype littermate control (*n* = 8) mice. For lights on/off and per hour of recording, data on minutes of wake (panels **A**, **B**), and total (panels **C**, **D**), NREM (panels **E**, **F**) and REM (panels **G**, **H**) sleep are summarized. Consistent with a normalization of the sleep/wake phenotype, we find no meaningful differences between *Homer1a −/−; mGluR(F-R)* and wildtype control mice in the amounts of sleep/wake during lights off. Data presented as mean±SE.

**Figure 8:**
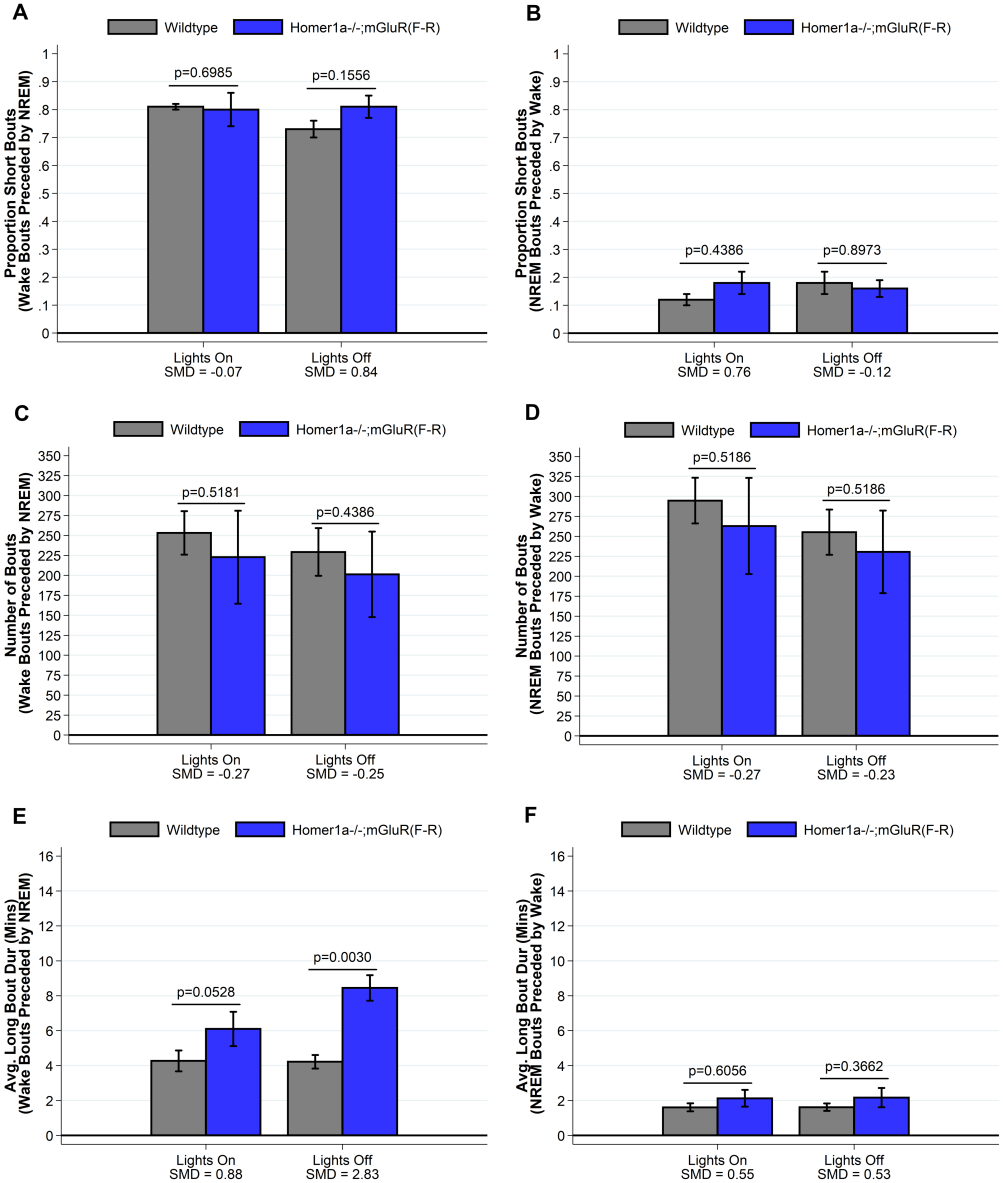
Sleep and wake phenotypes estimated from spike and slab approach in Homer1a −/−; mGluR(F-R) (*n* = 6) and wildtype littermate control (*n* = 8) mice. Phenotypes describing the number (panel **A**), proportion of short (panel **C**), and duration of long (panel **E**) wake bouts preceded by NREM (e.g., primary wake bouts) and number (panel **B**), proportion of short (panel **D**), and duration of long (panel **F**) NREM bouts preceded by wake (e.g., primary sleep bouts) are illustrated in *Homer1a −/−; mGluR(F-R)* and wildtype mice. Consistent with a normalization or rescue of the sleep/wake phenotype, we see that *Homer1a −/−; mGluR(F-R)* mice have a greater duration of long wake bouts preceded by NREM during lights off, with a consistent (but smaller magnitude) greater duration during lights on. Other traits are similar in mutant and wildtype mice. Data presented as mean±SE.

To establish whether the reduced ability to sustain wakefulness observed in *Homer1a* knock-out mice (Naidoo et al., 2012) was in part downstream of a Pin1 mechanism, we crossed Homer1a null mice with mGluR(F-R) mice to generate the double mutant Homer1a−/−;mGluR(F-R). These mice carry both the *Homer1a* knock-out mutation and a mutation preventing binding of Homer to mGluR5. While we observed no differences in sleep and wake characteristics using traditional summary measures (see Figure 7 and Supplementary Table S5), when evaluating the spike-and-slab phenotypes (see Figure 8 and Supplementary Table S6), we observed significantly *longer* duration of long bouts of wake preceded by NREM among the Homer1a−/−;mGluR(F-R) mice compared to littermate controls (SMD = 2.83; *p* = 0.003; Figure 8E). Consistent with this result, the empirical Q-Q plot comparing the distribution of the lengths of bouts of wake preceded by NREM supports significantly longer bouts among the Homer1a−/−;mGluR(F-R) mice (see Figure 4C). Similar evidence of increased long bouts of wake preceded by NREM in the Homer1a−/−;mGluR(F-R) was observed in the lights on period (SMD = 0.88, *p* = 0.053; Figure 8E) and over the full 24-h of recording (SMD = 1.80, *p* = 0.007; Supplementary Table S6). In addition, there was evidence of increased duration of long bouts of wake preceded by REM in the Homer1a−/−;mGluR(F-R) mice during lights on (SMD = 1.87; *p* = 0.010; Supplementary Table S6). The power density in the double mutant mice is higher in NREM and REM at frequencies greater than 20 Hz in the beta range (see Supplementary Figure S2). During wake there was reduction in power density in theta around 5-9 Hz, but increased power density in alpha around 11-13 Hz, sigma 15-18 Hz, and in frequencies greater than 20 Hz in the mutant (Supplementary Figure S2). Overall, there is evidence that the previously observed reduced ability to maintain wakefulness in the *Homer1a* knock-out and mGluR(TS-AA) strains may be improved in the double mutant.

### Assessment of Pin1, Homer1a and mGluR phosphorylation status in wildtype and mGluR(TS-AA) mice

**Figure 9:**
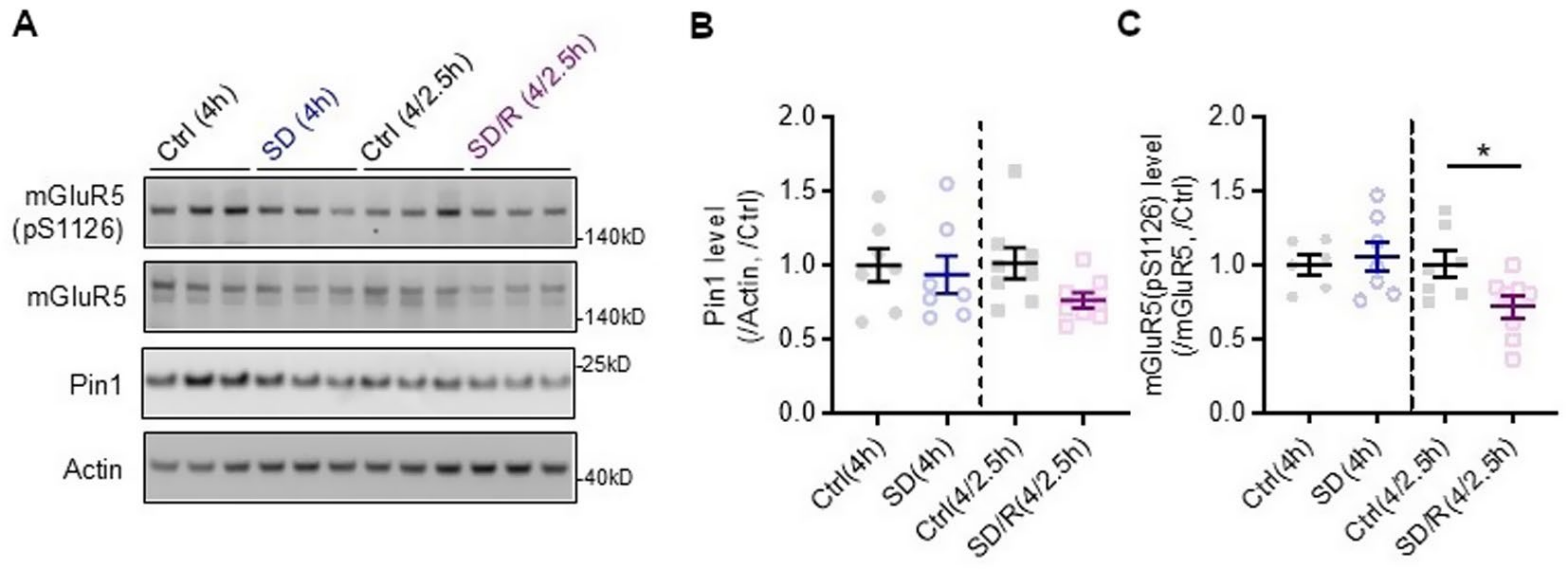
Representative blots **(A)** and quantification of Pin1 **(B)** and mGluR5(pS1126) **(C)** levels in cortical lysates of wildtype mice after sleep deprivation (SD), recovery sleep (R) and undisturbed diurnal controls (Ctrl). Pin1 protein expression is not changed by sleep deprivation. Phosphorylated mGluR5 is decreased with recovery sleep. mGluR5(pS1126)SD/R(4/2.5h) versus Ctrl(4/2.5h), *p = 0.047 one-way ANOVA followed by Bonferroni post hoc test; *n* ≥ 6 mice per group.

**Figure 10:**
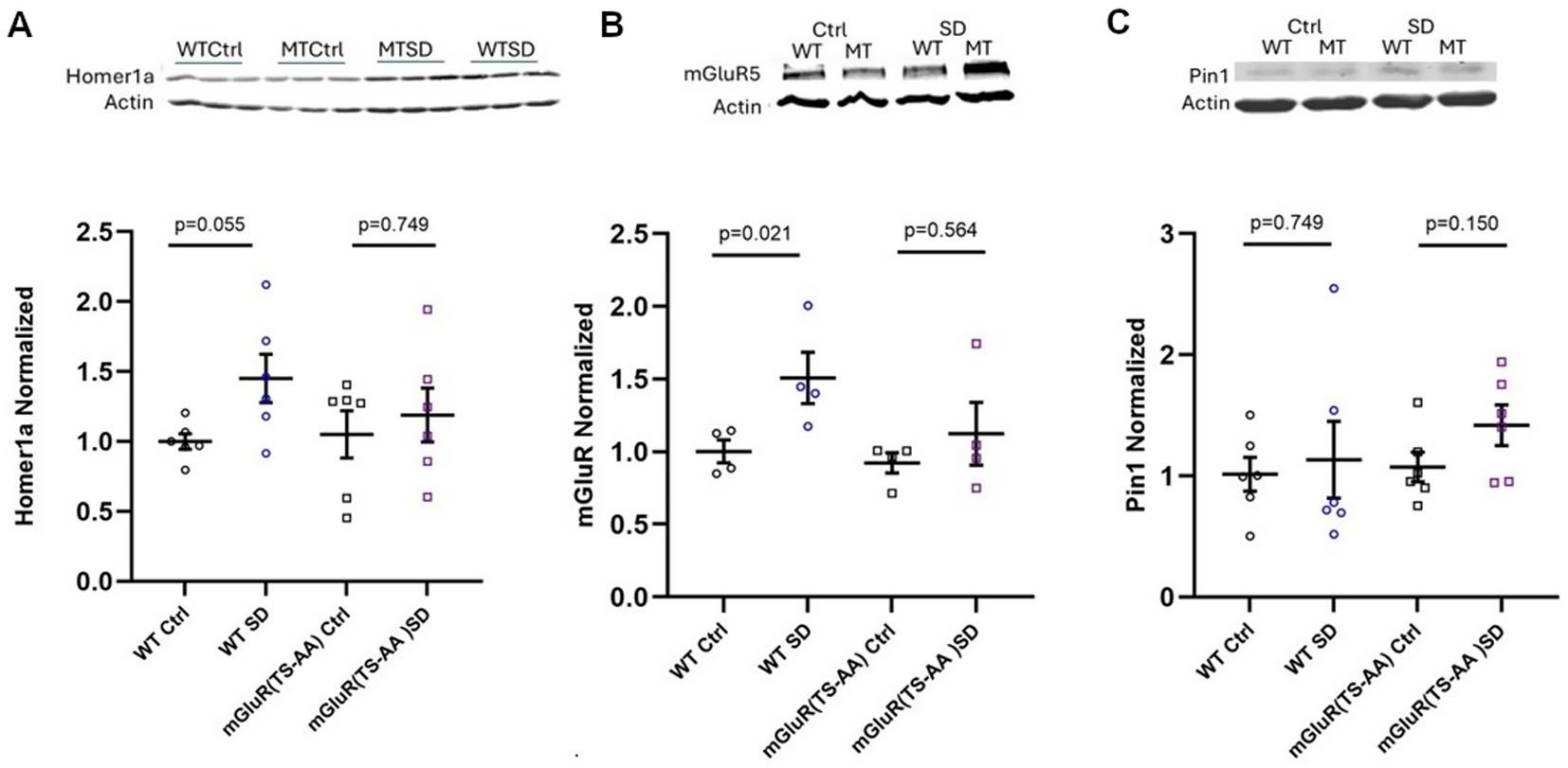
Quantification of Homer1a **(A)**, mGluR5 **(B)** and Pin1 **(C)** in cortical lysates of mGluR(TS-AA) mice following sleep deprivation (SD). Homer1a and mGluR5 are generally increased with SD in wildtype littermates, compared to no change in mGluR(TS-AA) knock-in mice. Pin1 appears unchanged with state in both wildtype and mGluR(TS-AA) mice. Representative western images shown above graphs; *n* = 4 mice per group for mGluR, *n* = 6 mice per group for Homer1a and Pin1.

Having established that the mGluR(TS-AA) mice display a sleep/wake phenotype like the *Homer1a* knock-out mice, we wanted to determine how Homer1a, Pin1 and mGluR changed in these mice during sleep deprivation and recovery sleep. We recently recapitulated previous findings in wildtype mice that Homer1a increases with sleep deprivation (Lin et al., 2021). Here, we tested whether Pin1 and phosphorylated mGluR5 are altered with sleep deprivation and recovery sleep (Figure 9) and measured the protein expression of Homer1a, Pin1 and mGluR in the mGluR(TS-AA) mutant mice (Figure 10).

#### Pin1 protein expression does not change with sleep deprivation in wildtype mice

To determine whether Pin1 and phosphorylated mGluR, which is expected to drive Pin1 binding, are altered with sleep deprivation or recovery sleep, we used western blot analyses of cerebral cortex lysates to assess their protein expression (see Figure 9). We observed no alterations in Pin1 protein levels following sleep deprivation (Figure 9B). While not statistically significant, Pin1 levels were reduced during recovery sleep. Similarly, we observed no change in mGluR5 with sleep deprivation, but did observe a significant decrease in phosphorylated mGluR5 with recovery sleep in wildtype mice (Figure 9C). Given the observed decrease in phosphorylated mGluR5, we measured the kinase, phospho-Erk, which is known to phosphorylate mGluR5 at this site (Park et al., 2013). Phospho-Erk was not changed with sleep deprivation or recovery sleep (Supplementary Figure S3).

#### Homer1a protein and Pin1 are not increased in mGluR(TS-AA) mutant mice with SD

To understand the molecular response to sleep deprivation in mGluR(TS-AA) mutants, we measured Homer1a, mGluR and Pin1 protein expression in cortical lysates of mGluR(TS-AA) and wildtype littermate mice (see Figure 10). Here, as previously reported (Naidoo et al., 2012), we found that Homer1a was generally increased in the cortical tissue of the wildtype littermate mice with 3 h of sleep deprivation (*p* = 0.055; Figure 10A). While in previous studies we had reported increased Homer1a mRNA expression in the piriform and cingulate cortices as well as the claustrum of wildtype mice with 1 h of sleep deprivation (Zhu et al., 2020), it was not assessed in this study. We observed no increase in Homer1a protein in cerebral cortex tissue of the mGluR(TS-AA) mice with 3 h of sleep deprivation (*p* = 0.749; see Figure 10A). We observed an increase in mGluR in wildtype cortical tissue (*p* = 0.021), but no change in that from the mutant mice with sleep deprivation (*p* = 0.564) (see Figure 10B). Pin1 protein levels were not significantly altered in either the wildtype littermates or the mGluR(TS-AA) mutant with sleep deprivation, but there was a trend towards higher in the mGluR(TS-AA) mutant (*p* = 0.150; Figure 10C). This data indicates that the Homer1a response in the mGluR(TS-AA) mutant is not altered and that the observed change in wake behavior observed in these mutant mice is not likely due to altered expression of Pin1.

## Discussion

We set out in this study to understand the mechanism by which the Homer1a interaction with mGluR contributes to the maintenance of wakefulness. Altogether, our data indicate that the Pin1 binding to mGluR in the presence of Homer1a underlies wake maintenance and that altering this site changes sleep/wake behavior in knock-in mice, consistent with the Homer1a knock-out phenotype. It is likely that other binding partners of Homer and mGluR could also affect this behavior.

First, we evaluated the mGluR(TS-AA) knock-in mouse model, which has altered binding sites for the prolyl isomerase Pin1. In wildtype mice, Pin1 accelerates rotation of the phosphorylated S/T–P bond in target proteins and acts as a molecular switch (Park et al., 2013). Homer1a, induced in response to neuronal activity, disrupts Homer cross-linking and potentiates Pin1-mediated isomerization of phosphorylated mGluR promoting activity. The combined phosphorylation of T1123 and S1126 purportedly increases Homer1 EVH1 binding affinity by 40-fold and assures that Pin1 action is conditional upon the presence of Homer1a at the synapse (Park et al., 2013). It has been suggested that the increased affinity may also serve to concentrate Homer1a at activated synapses (Park et al., 2013). Thus, the loss of the Pin1 binding site in the mGluR(TS-AA) mice would result in reduced Homer1a binding affinity for mGluR, as well as less Homer1a at the synapse, which was predicted to result in a sleep phenotype similar to that observed with loss of Homer1a. Consistent with this prediction, mGluR(TS-AA) mice show a sleep/wake phenotype similar to that of the *Homer1a* knock-out. Specifically, we had previously demonstrated that knock-out of the dominant negative form of Homer, *Homer1a*, resulted in a phenotype of reduced wakefulness due to the inability of the animals to sustain long bouts of wake during the lights off period (Naidoo et al., 2012). Similarly, we find that the mGluR(TS-AA) mice have a greater proportion of short bouts of wake preceded by NREM during lights off; the empirical Q-Q plots of these bouts show that the mGluR(TS-AA) mice have much shorter bouts of wakefulness than the wildtype mice, entirely aligned with our previously published data in *Homer1a* knock-outs (Naidoo et al., 2012). Other characteristics during lights off are consistent with this reduced ability to maintain wakefulness (e.g., reduced wake and increased sleep times, as well as a smaller proportion of short bouts of NREM preceded by wake). Additionally, as observed in *Homer1a* knock-outs (Naidoo et al., 2012), we also find that the mGluR(TS-AA) mice had more REM sleep during the active lights off period, suggesting a role for the Pin1-mGluR-Homer1a interaction in REM sleep regulation.

Previous transcriptomic data (Mackiewicz et al., 2007; Maret et al., 2007) and current biochemical analyses indicate that Homer1a is increased with sleep deprivation, allowing the disruption of Homer1 cross-linking and Pin1 catalysis. As expected, we found that Homer1a was generally increased with sleep deprivation in the wildtype littermate mice, but not in the mGluR(TS-AA) mice; this may partly explain the reduced wake phenotype observed in these mice. As Pin1 binds to the pSer/Thr-Pro motifs of proteins and regulates their gene transcription by altering the stability, subcellular localization, protein–protein interactions, and protein-DNA/RNA interactions (Xu and Manley, 2007), it is likely that *Homer* gene expression is affected by the altered Pin1 binding in the mGluR(TS-AA) mutant.

We also examined whether genetically altering the Homer binding site, P–P-X-X-F, on mGluR5 would alter the sleep/wake phenotype by studying an mGluR(F-R) knock-in mouse. Within our sample of mice, we found that replacing phenylalanine with arginine did not significantly modify sleep/wake behavior when compared to littermate controls, with generally small/moderate differences between groups. The Pin1 binding site remains unaltered in the mGluR(F-R) mice, suggesting that Pin1 isomerization of the S/T–P bond in mGluR5 drives activity. We did, however, observe an increase in REM sleep in the mGluR(F-R) mice during the lights on period. A transient increase in dopamine in the basolateral amygdala is known to promote REM (Hasegawa et al., 2022). In the absence of any Homer binding in the mGluR(F-R) mutant mice, the mGluR-Pin1 interaction is constitutive and this interaction is known to mediate a dopamine dependent plasticity (Park et al., 2013), which is a likely mechanism for the observed increase in REM in these mice.

We then tested whether Pin1 action drove activity by crossing *Homer1a* null mice with mGluR(F-R) mice to generate double mutants lacking both *Homer1a* and the Homer-mGluR binding site. Interestingly, these mice had evidence of increased duration of long wake bouts, rather than an inability to maintain wakefulness, with other sleep/wake characteristics similar to wildtype mice. These mice also displayed altered power density during wake, NREM and REM. In particular the double mutants had significantly reduced theta, but increased alpha and sigma than the wildtype littermate mice during wake. Theta usually increases with wake and has been described to be a wake EEG marker of sleep need (Cajochen et al., 1995; Vyazovskiy and Tobler, 2005; Hung et al., 2013). The decrease in theta during wake in the double mutant suggests reduced sleep drive. Wake EEG theta and alpha also depend on the quality of wakefulness (Cajochen et al., 1995). Alpha activity is typically associated with relaxed wakefulness (Cantero et al., 2002). Increased sigma activity during wake is thought to represent a transitory state into sleep (Morikawa et al., 2002) Together, the spike-and-slab and power spectral analyses in the Homer1a−/−;mGluR(F-R) mice both suggest that the Pin1 mechanism may be sufficient to rescue or reverse the wake phenotype observed in the *Homer1a* knock-out. Consistent with these results, in previous studies we had shown that heterozygous Homer1a knock-out mice had a normal sleep/wake phenotype (Naidoo et al., 2012).

Our biochemical analyses indicated that sleep deprivation did not alter Pin1 protein expression in either the wildtype or mutant mGluR(TS-AA) mice, while mGluR5 protein levels increased with sleep deprivation in wildtype cortical tissue. While inconsistent with earlier data showing no change in mGluR with sleep deprivation (Lin et al., 2021), this prior study had a 4-h duration of sleep deprivation compared to only 3 h in the present data; the altered protein levels in this study could be due to an early increase in mGluR protein. However, we did find that phosphorylation of mGluR5 at S1126 decreased with recovery sleep, which may be attributed to reduced neuronal activity. We had tested whether altered kinase activity contributed to this observation, but found that phospho-Erk, which phosphorylates mGluR5 at this site, was not changed with sleep deprivation or recovery sleep. Previously, we reported that Shank3 decreases with sleep deprivation and, paralleling Homer1a, returns to baseline with recovery sleep (Lin et al., 2021). Whether this influences sleep/wake behavior in this study is unknown, but Shank3 mutant mice have been shown to sleep less than wildtype mice during the active lights off period (Ingiosi et al., 2019). We did find that Homer1a was not induced by sleep deprivation in the mGluR(TS-AA) mice, suggesting that this could be an additional contributing factor to the observed sleep/wake phenotype in the knock-in mice. Upregulation of Homer1a during either wake or sleep deprivation is expected to enhance binding of Pin1 to mGluR, contributing to the maintenance of wakefulness. Consistent with this idea, we have previously shown that CREB alpha delta mutant mouse that fails to up-regulate Homer1a with sleep deprivation also displays a reduced wake phenotype similar to the *Homer1a* knock-out and the mGluR(TS-AA) mice (Graves et al., 2003; Naidoo et al., 2012). A limitation of this study was the lack of biochemical data from the claustrum, which has been shown to be a region within which Homer1a is rapidly induced by sleep loss (Zhu et al., 2020). This will be addressed in a future study.

The strength of our study lies in the use of mutant mice to directly examine and illustrate, in part, a mechanism underlying the behavioral phenotype of the Homer1a knock-out mice. There are also a few limitations. Consistent with the original *Homer1a* knock-out study that served as the foundation for our hypotheses, the study was conducted only in male mice. Moreover, our sample size was relatively small and had >80% power only for large SMD (between 1.5–1.7, depending on the genotype; see *Methods*). Thus, non-statistically significant results for smaller effect sizes should be interpreted with caution as they may represent false negative associations. For this reason, as recommended by recent articles in the statistical literature (Wasserstein et al., 2019) and from Editors of respiratory, sleep and critical care journals (Lederer et al., 2019), in addition to reporting statistical significance results are presented along with standardized effect sizes, with 0.2, 0.5 and 0.8 representing small, medium and large effects (Cohen, 1988). Overall, our data leads us to speculate that the mGluR-Pin1 effect mediates a form of D1R dopamine or perhaps TrkB metaplasticity that stabilizes excitatory ensemble formation. This is different than previously published data indicating that Homer1a acts to weaken synapses (Diering et al., 2017).

## Conclusion

Altogether, our data indicate that state-dependent Pin1 binding to mGluR influences sleep/wake behavior and that altering this site alters sleep behavior in knock-in mice. Pin1 binding to mGluR during periods of prolonged waking, when Homer1a expression is expected to be at its highest, amplifies mGluR activity allowing longer bouts of sustained wakefulness. It is likely that other binding partners of Homer and mGluR also affect this behavior.

## Data Availability

The original contributions presented in the study are included in the article/Supplementary material, further inquiries can be directed to the corresponding author.

## Supplementary material

SUPPLEMENTARY FIGURE S1 Spectral power analysis comparing mGluR(TS-AA) (*n*=7) and wildtype littermate control (*n*=7) mice. The power spectral curves illustrating the power density as a percentage of average power from 0–30Hz are illustrated in mGluR(TS-AA) mice and wildtype littermate control mice. Overall, we observed little differences in the power curves between the two genotypes, with only a single *p* < 0.05 at around 9Hz during wake in the lights off period. Data presented as mean ± SE; **p* < 0.05.

SUPPLEMENTARY FIGURE S2 Spectral power analysis comparing Homer1a −/−; mGluR(F-R) (*n*=6) and wildtype littermate control (*n*=8) mice. The power spectral curves illustrating the power density as a percentage of average power from 0–30Hz are illustrated in Homer1a −/−; mGluR(F-R) mice and wildtype littermate control mice. The power density in the mutant mice is higher in both NREM and REM at frequencies greater than 20Hz in the beta range. Changes were also seen in theta around 5–8Hz, alpha around 11–12hz, and sigma 15–18 Hz during wake. Data presented as mean±SE; **p*<0.05.

SUPPLEMENTARY FIGURE S3 Representative blots and quantification of p-Erk levels in cortical lysates of wildtype mice after sleep deprivation (SD), recovery sleep (R) and undisturbed diurnal controls (Ctrl). p-Erk protein expression is not changed by sleep deprivation or recovery sleep; *n* ≥ 6 mice per group.
