# Supplementary figures and images for: Mutant mouse models implicate a role for mGluR1/5, prolyl isomerase (Pin1) and Homer1a interactions in wakefulness
Source: Front Neurosci. 2025 Sep 11;19:1572258. doi: 10.3389/fnins.2025.1572258 (PMC12460416; doi:10.3389/fnins.2025.1572258)

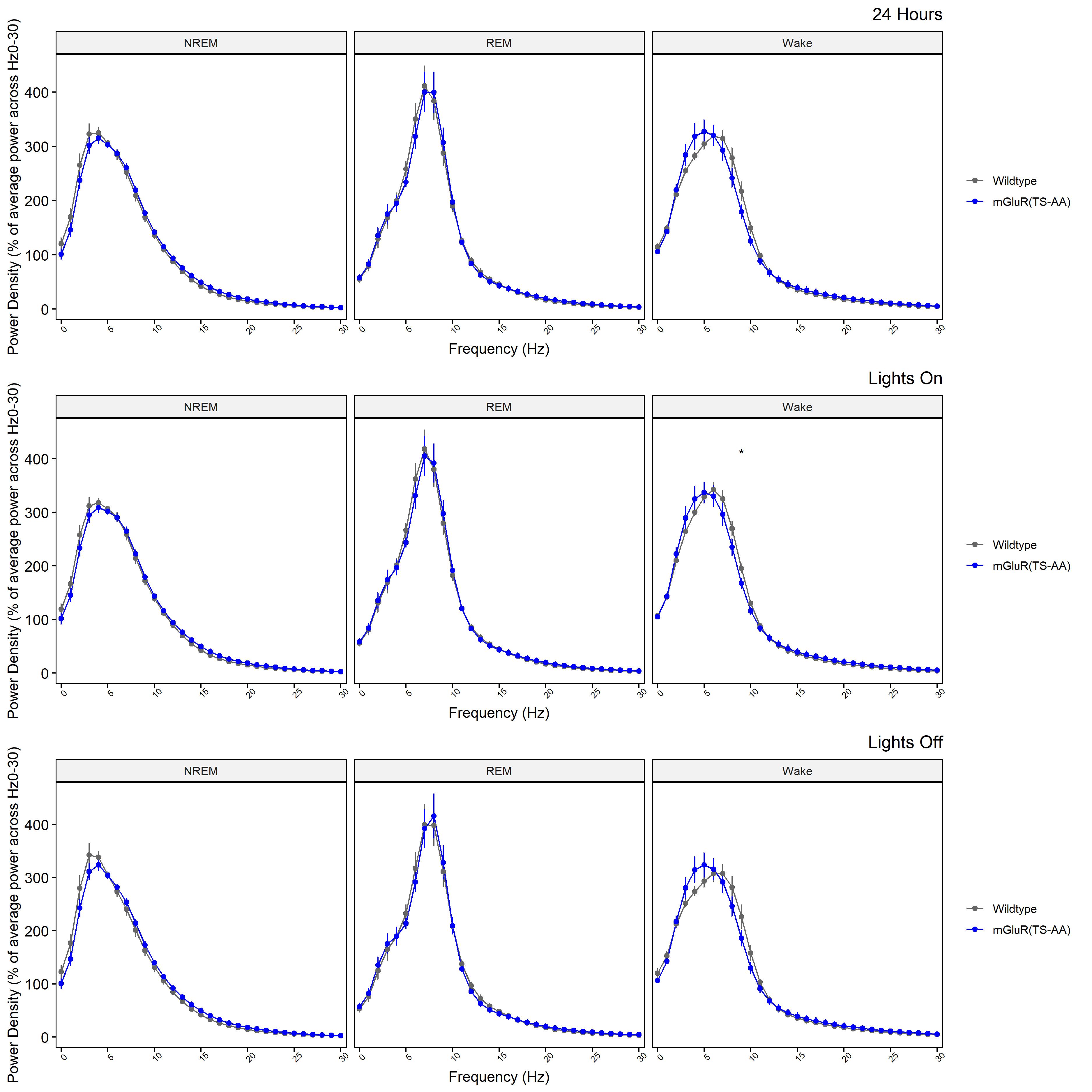

Supplement: SUPPLEMENTARY FIGURE S1 — Spectral power analysis comparing mGluR(TS-AA) (n=7) and wildtype littermate control (n=7) mice. The power spectral curves illustrating the power density as a percentage of average power from 0–30Hz are illustrated in mGluR(TS-AA) mice and wildtype littermate control mice. Overall, we observed little differences in the power curves between the two genotypes, with only a single p < 0.05 at around 9Hz during wake in the lights off period. Data presented as mean ± SE; *p < 0.05. [file Image_1.jpeg]

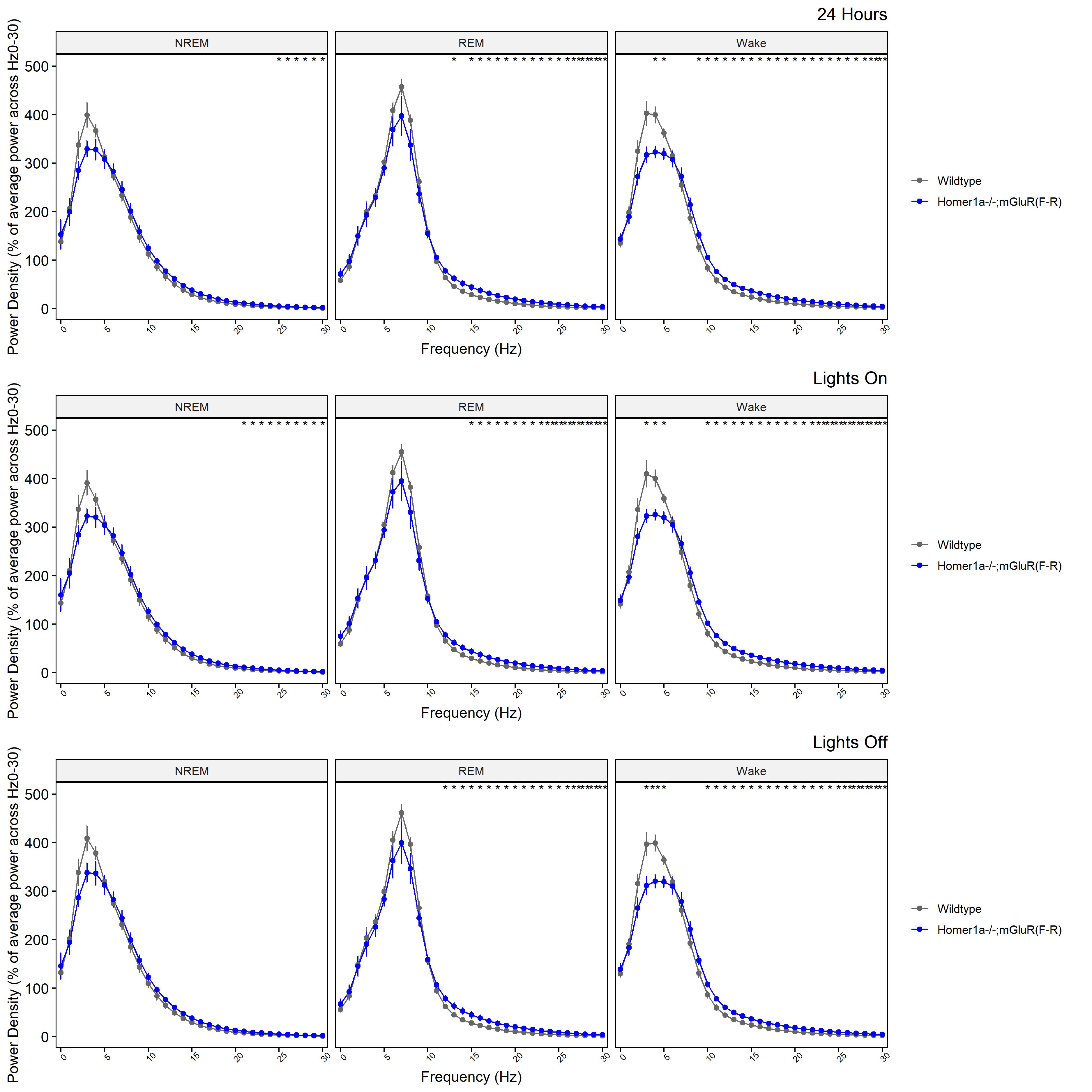

Supplement: SUPPLEMENTARY FIGURE S2 — Spectral power analysis comparing Homer1a −/−; mGluR(F-R) (n=6) and wildtype littermate control (n=8) mice. The power spectral curves illustrating the power density as a percentage of average power from 0–30Hz are illustrated in Homer1a −/−; mGluR(F-R) mice and wildtype littermate control mice. The power density in the mutant mice is higher in both NREM and REM at frequencies greater than 20Hz in the beta range. Changes were also seen in theta around 5–8Hz, alpha around 11–12hz, and sigma 15–18 Hz during wake. Data presented as mean±SE; *p<0.05. [file Image_2.jpeg]

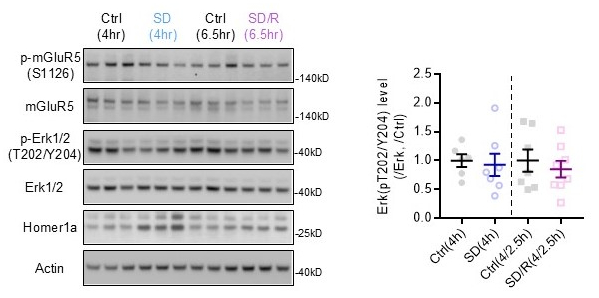

Supplement: SUPPLEMENTARY FIGURE S3 — Representative blots and quantification of p-Erk levels in cortical lysates of wildtype mice after sleep deprivation (SD), recovery sleep (R) and undisturbed diurnal controls (Ctrl). p-Erk protein expression is not changed by sleep deprivation or recovery sleep; n ≥ 6 mice per group. [file Image_3.jpeg]
